# An Active Learning Approach for Rapid Characterization of Endothelial Cells in Human Tumors

**DOI:** 10.1371/journal.pone.0090495

**Published:** 2014-03-06

**Authors:** Raghav K. Padmanabhan, Vinay H. Somasundar, Sandra D. Griffith, Jianliang Zhu, Drew Samoyedny, Kay See Tan, Jiahao Hu, Xuejun Liao, Lawrence Carin, Sam S. Yoon, Keith T. Flaherty, Robert S. DiPaola, Daniel F. Heitjan, Priti Lal, Michael D. Feldman, Badrinath Roysam, William M. F. Lee

**Affiliations:** 1 Department of Electrical and Computer Engineering, University of Houston, Houston, Texas, United States of America; 2 Department of Quantitative Health Sciences, Cleveland Clinic, Cleveland, Ohio, United States of America; 3 Department of Medicine, University of Pennsylvania, Philadelphia, Pennsylvania, United States of America; 4 Department of Biostatistics and Epidemiology, Center for Clinical Epidemiology and Biostatistics, University of Pennsylvania, Philadelphia, Pennsylvania, United States of America; 5 Department of Electrical and Computer Engineering, Duke University, Durham, North Carolina, United States of America; 6 Department of Surgery, Memorial Sloan-Kettering Cancer Center, New York, New York, United States of America; 7 Department of Medicine, Harvard Medical School, Boston, Massachusetts, United States of America; 8 Department of Medicine, Hematology/Oncology, MGH, Boston, Massachusetts, United States of America; 9 The Cancer Institute of New Jersey, New Brunswick, New Jersey, United States of America; 10 UMDNJ-Robert Wood Johnson Medical School, New Brunswick, New Jersey, United States of America; 11 Abramson Cancer Center, Perelman School of Medicine, University of Pennsylvania, Philadelphia, Pennsylvania, United States of America; 12 Department of Pathology and Laboratory Medicine, University of Pennsylvania, Philadelphia, Pennsylvania, United States of America; University of Pécs Medical School, Hungary

## Abstract

Currently, no available pathological or molecular measures of tumor angiogenesis predict response to antiangiogenic therapies used in clinical practice. Recognizing that tumor endothelial cells (EC) and EC activation and survival signaling are the direct targets of these therapies, we sought to develop an automated platform for quantifying activity of critical signaling pathways and other biological events in EC of patient tumors by histopathology. Computer image analysis of EC in highly heterogeneous human tumors by a statistical classifier trained using examples selected by human experts performed poorly due to subjectivity and selection bias. We hypothesized that the analysis can be optimized by a more *active* process to aid experts in identifying informative training examples. To test this hypothesis, we incorporated a novel active learning (AL) algorithm into FARSIGHT image analysis software that aids the expert by seeking out informative examples for the operator to label. The resulting FARSIGHT-AL system identified EC with specificity and sensitivity consistently greater than 0.9 and outperformed traditional supervised classification algorithms. The system modeled individual operator preferences and generated reproducible results. Using the results of EC classification, we also quantified proliferation (Ki67) and activity in important signal transduction pathways (MAP kinase, STAT3) in immunostained human clear cell renal cell carcinoma and other tumors. FARSIGHT-AL enables characterization of EC in conventionally preserved human tumors in a more automated process suitable for testing and validating in clinical trials. The results of our study support a unique opportunity for quantifying angiogenesis in a manner that can now be tested for its ability to identify novel predictive and response biomarkers.

## Introduction

Cancers are a complex community of different cell types with the transformed tumor cell population usually receiving primary attention. Cells of the tumor stroma, often considered secondary, provide essential support for tumor cells and are increasingly recognized for their contributions to the malignant behavior of cancers [1]. Among stromal cells, the importance of endothelial cells (EC) is widely recognized, which has led to the development [2], [3] and clinical adoption [4], [5] of treatments targeting tumor EC as a way of controlling tumor growth and spread. The pathophysiologic and therapeutic significance of EC in cancer biology suggests the need to study and characterize EC in patient tumors. However, there have been few efforts to study these cells in human cancers beyond assessing the density and limited characteristics of microvessels [6], [7], [8]. Low numbers, inconspicuous and pleiomorphic appearance and dispersal throughout the disorganized architecture of tumors make systematic visual identification of EC in histopathology very challenging. Biomarker staining can facilitate EC identification but adds to the complexity of visual inputs that observers must process (Fig. S1). Given the necessary expertise, time, and labor, visual analysis of cancer EC is not practical on a clinical scale.

Based on prior studies in mouse tumor models [9], [10], we wanted to examine biological events, such as proliferation and activation of signaling pathways, in EC of human tumors to understand their biology in real cancers and identify potential metrics of tumor angiogenic activity. EC in human renal cell carcinomas (RCC) were of particular interest because deregulated angiogenesis features prominently in the pathogenesis of these tumors [11], and anti-angiogenic agents are preferred treatment for patients with metastatic RCC [12]. The goal of characterizing RCC EC in patients entered in therapeutic clinical trials meant that tumors from many patients would need to be studied, and a computer-assisted approach to analysis would be needed. To this end, we developed a novel image analysis platform to perform selective molecular imaging of patient tumor tissue, accurately delineating structures and reliably identifying cell types of interest using the FARSIGHT software platform [13].

Performing comprehensive, cytometric analysis of EC from tumor images is predicated on the identification of individual cells present in the tumor and their classification into cell types of interest. We developed a hybrid nuclear segmentation algorithm (Methods Section) that delineates individual nuclei in the image resulting in a label map that defines the presence of all nuclei. The label map allows the computation of quantitative measurements (referred to as features) that describe the shape, size, texture and intensity of associated biomarkers for individual nuclei. These features are provided as an input to a classification algorithm that learns different classes of cells from the training examples and labels provided by an expert. In the traditional classification paradigm, the user sifts through a pool of unlabeled cells and provides class labels (e.g. endothelial cell or tumor cell) for a subset. The labeled subset is provided as an input to the algorithm which uses these features and labels to construct a mathematical model and classify all the cells present in the dataset. However, human trainers can spend considerable effort providing many uninformative examples, because they cannot judge the quantitative contribution of an example to the learning task. Also, when the analysis is performed at the scale of clinical trials with hundreds of thousands of cells, selecting useful examples can be an effort-intensive proposition. Unaided, they can introduce subjectivity and selection bias into the learning process which might hurt classification performance. In order to make the training objective and minimize the training effort, we adapted and improved a novel *active* learning algorithm developed originally for Unexploded Ordnance Cleanup [14].

The fundamental idea behind active learning (AL) is that not all training examples are equally useful for classification. Based on what we know about the cells by observing the labels for a few training examples, labels of certain examples are more useful than others. By focusing on these examples iteratively, the algorithm can learn the problem more quickly and classify the data more accurately. AL algorithms sequentially select the most informative cells from the large unlabeled pool and query the user for their labels. Based on how the notion of information is defined, several active learning methods have been proposed. One of the most popular frameworks is the Uncertainty Sampling where the algorithm queries for examples that it is most uncertain about. It is commonly used with margin based classifiers like SVM [15] and also with probabilistic classifiers where the notion of uncertainity is easily defined in terms of probabilities of the class. Query by Committee methods [16], [17] use an ensemble of classfiers and query training examples that have the maximum disagreement in their labeling by the committee members. Expected Error Reduction methods [18], [19] query those examples that reduce the future generalization error of the classifier and are computationally expensive. The proposed approach falls under the category of Variance Reduction methods where examples that reduce the variance and thereby the uncertainty in the parameter estimates are chosen for labeling and information is quantified as a derived quantity of the Fisher Information Matrix (Methods Section). Active learning methods have found success in several domains like text classification [15], [17], microarray analysis [20], information retrieval [21], recommender systems [22] etc. Active learning methods have also been applied in the field of histopathology with considerable sucess [23], [24].

Traditionally, supervised classification algorithms also require the users to select the best set of features that will help differentiate between different cells of interest. The more discriminative the set of features selected, the better the classification accuracy. However, most feature selection algorithms require a representative training set apropri to select the best set of features for classification. Other feature reduction methods involve exploiting the information in the eigenstructure of the data and project the original data into a latent space where the new features are linear/non-linear combination of original features. Although effective, the transparency of features is lost when latent space methods are used and additional effort is required to interpret these results. Most feature selection algorithms require the user to perform offline analysis. To select the important features on the fly and obviate the offline feature selection process, we improved the active learning algorithm to automatically select the important features for classification also, thereby freeing the user from expending additional effort for deciding the relevant features for different cell classification problems. The complex mathematics of the proposed algorithm (see Methods for details) is hidden from the user and it is integrated into the FARSIGHT software system with graphical user interfaces that constantly update the user about the progress of classification and the parameters being used, making the classification process transparent and practically usable. The results of our study indicate that the proposed approach enables characterization of EC in human tumors that can be tested and validated in clinical trials.

## Results

### Endothelial cell identification in human tumor histopathology

Our study requires analysis of vascular EC in patient ccRCC through automated analysis of large batches of multi-spectral images of tumor slides immunostained for CD34 to reveal EC (250–500 images/batch are common). We previously developed FARSIGHT to analyze images and classify carcinoma cells based on morphometric characteristics and association with epithelial cytokeratin or other biomarker staining [25]. When we used similar association rules to classify EC on a subset of ccRCC images, the resulting classification had many errors when compared to expert human EC classification of the same images. To improve automated EC classification, we incorporated supervised machine learning classifier algorithms (Kernel Partial Least Squares, KPLS [26]) into FARSIGHT and developed protocols for providing examples of EC and non-EC for training. EC classification improved, but the primary goal of high specificity of EC classification (>0.95) was achieved only in very few images, and these tended to be the actual images used for training or different images from the same or similar tumors. It also came with considerable sacrifice in sensitivity. Results did not improve substantially with the addition of carbonic anhydrase (CA) IX and smooth muscle actin (SMA) immunostaining to highlight tumor cells and pericytes, respectively, for exclusion during EC classification. More training with additional examples of EC and non-EC in the same or new images only improved classification marginally or not at all. On closer inspection, the poor performance of these algorithms was discovered to be a result of the immense diversity in both CD34 biomarker staining patterns as well as features of the cells in the large image set, all part of the inescapable heterogeneity of patient tumor samples. The training set, by necessity, identified only a minor sub-group of EC that may not exemplify the critical features or criteria for accurate classification and, hence, performed poorly when applied on the entire dataset. Supervised machine learning algorithms are effective only when provided with labeled examples that capture all essential information about the data for which predictions are sought. As training samples selected exclusively by humans did not produce a classifier that could classify EC in RCC reliably, we hypothesized that performance would be optimized by incorporating a novel active learning algorithm based on the logistic regression classifier in FARSIGHT (FARSIGHT-AL).

**Figure 1:**
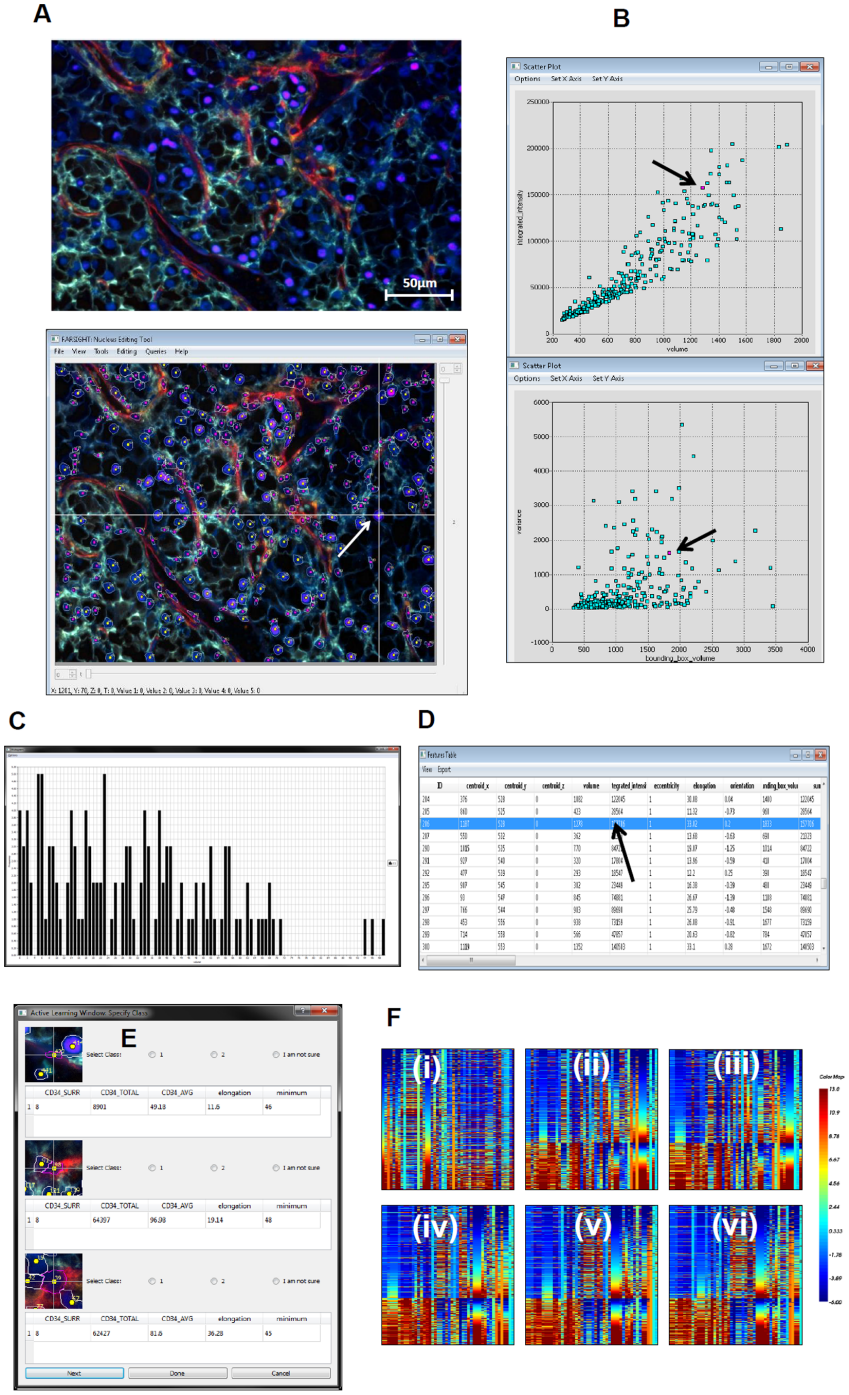
FARSIGHT–AL software interface. The FARSIGHT-AL software interface integrates multiple views of the image data in a linked manner. Image view (**A**) allows the user to adjust the complexity of visual input with respect to the number of biomarkers he/she wishes to view. It also shows the pre-algorithm mask image to illustarte the detection capability of the software. The Scatterplot view (**B**), Histogram view (**C**), and Table View (**D**) enable the user to visualize the data in ways intended to extract different types of information. All the views are hot-linked i.e., a cell selected in the image view is highlighted in the scatter plot and table views as well (indicated by arrows). FARSIGHT-AL query window (shown in (**E**)) displays the informative examples selected by the algorithm for labeling along with an image snapshot of the cell. The 5 most important features selected by the algorithm are also displayed to the user. The evolving heatmap (**F**) shows an emerging structure with the active learning iterations (from i-vi) that provide an indication of convergence of classification.

To teach FARSIGHT-AL to classify EC in a set of tumor images, the trainer provides one initial example of EC and non-EC nuclei. The system then displays a list of cell features that can be utilized for the current classification task. Since the algorithm automatically selects the important features, we initially performed a “blind” analysis where all the computed features (Table S1 in File S1) were provided as inputs to the algorithm. We refer to this mode of the algorithm as “auto-select” mode in contrast to “manual-select” mode where an expert personally selects the relevant features for classification. In our study, experts were trained pathologists and an analyst trained in EC analysis in human histopathology specimens. The algorithm searches the feature measurements of all segmented cells and presents the cells with features that result in the maximum information gain (computed via the Fisher information matrix) for the trainer to label. Based on the response of the trainer, the algorithm updates its parameters, selects the best set of features that explain the classification of the current labeled set and also computes the most informative cells for the user to label in the next iteration. After each iteration, the algorithm computes the increase in maximum information gain and automatically senses convergence of its evolving EC classification model when the increase in information gain attains a plateau. The algorithm also updates the user interface that displays a set of informative plots and heatmaps indicating the state of the algorithm. The interface also allows the user to explore the features of cells in different views allowing the user to explore the parameter space comprehensively (Fig. 1). Upon convergence, FARSIGHT-AL proceeds to classify all cells as EC or non-EC and overlays a class-specific color-coded dot on the cells for user verification. The EC classification model developed by FARSIGHT-AL using one set of images can be archived and used to classify EC in other images.

**Figure 2:**
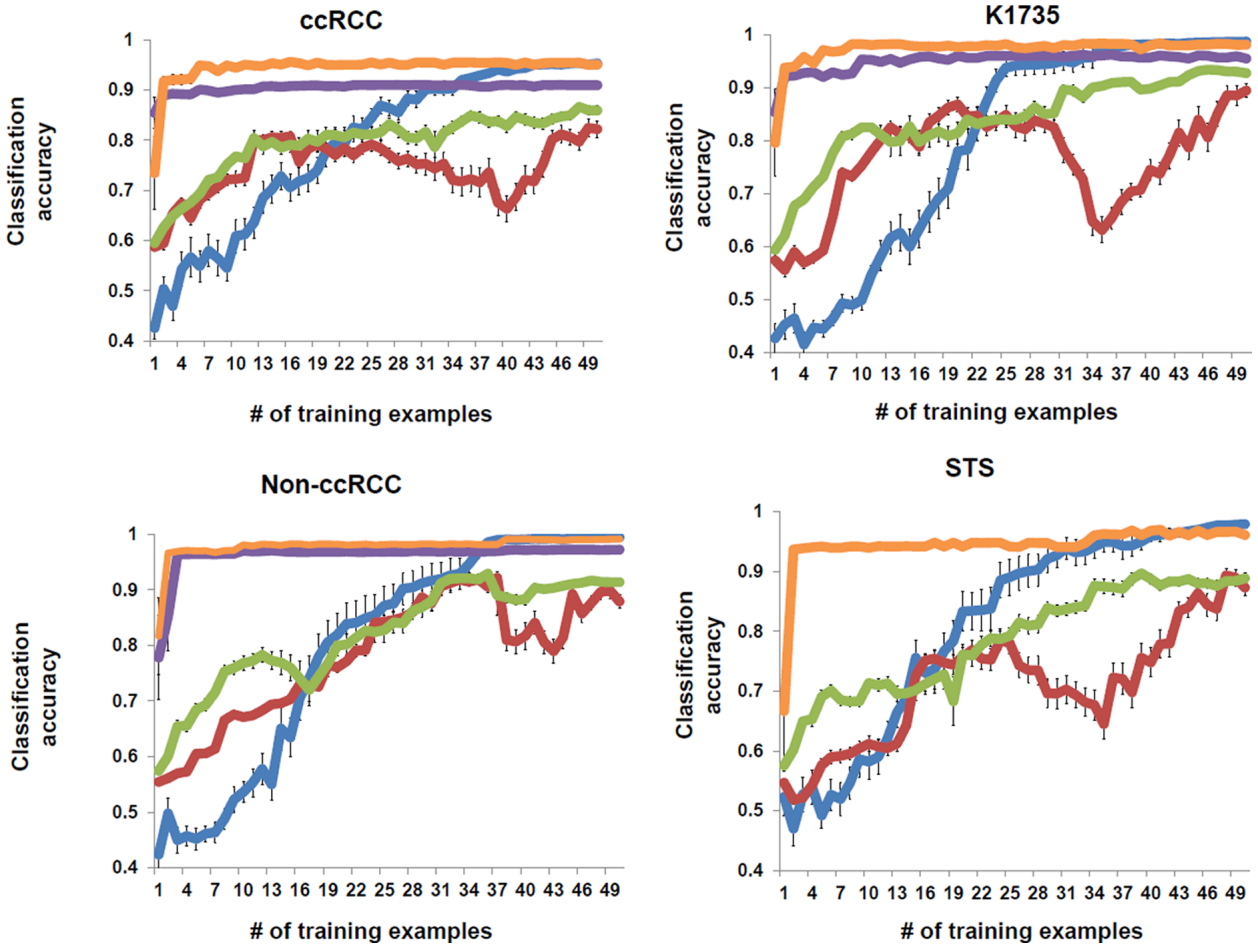
Comparison of FARSIGHT-AL performance with other feature selection algorithms. Mean classification accuracy of 25 independent simulations plotted as a function of number of training examples for differnet automated feature selection algorithms including FARSIGHT AL (blue lines) on four different datasets. FARSIGHT-AL selected 50 training examples sequentially based on the increase in information gain whereas logistic regression was used to classify examples after feature selection by PCA (green), T-Test (purple), MRMR (Orange). Standard Logistic regression (red) with no feature selection performs poorly compared to other algorithms. The bars indicate standard error of the mean of classification accuracy. For the STS dataset, the features chosen by MRMR and T-Test were identical which result in identical classifier performance indicated by the orange line.

We created a set of 20 images of stained ccRCC tumors from five different patients (ccRCC20 image set) with which to train and test the program (Fig. S1 shows a typical image analyzed). A total of 8,360 cells segmented by the FARSIGHT-AL nuclear segmentation algorithm were manually classified by a pathologist (trainer 2 in subsequent results) to train and evaluate the performance of the algorithm. 50 active learning iterations were performed to select the most informative cells from the unlabeled pool. The algorithm automatically weighted the features based on their discriminative capacity for the classification task and automatically set the weight of uninformative features to (nearly) zero, thereby nullifying their participation in the learning process. The performance of our algorithm was evaluated using classification accuracy as the metric and compared to that of the “standard” logistic regression classifier that chooses training examples randomly without any explicit criterion to select discriminative features. The proposed algorithm outperformed standard logistic regression and its performance was more reliable (Fig. 2 (A)). We also compared the performance of our algorithm with other feature selection algorithms: Prinicpal Component Analysis (PCA), Univariate Feature selection via t-test (T-Test) [27] and Minimum Redundancy-Maximum Relevance (MRMR) [28]. PCA uses the spectral information and captures the direction of maximum variance in the data by projecting the data on the principal components which are the eigen vectors of the covariance matrix of the data. The simple univariate feature selection method assumes that there is no interaction between the features and applies a univariate criterion i.e., the t-test on each feature and compares the p-value for each feature to determine its effectiveness in separating the classes. The MRMR feature selection algorithm uses mutual information criteria to compute the optimal set of features that are maximally different from each other but at the same time highly correlated with the classification variable. The comparison of performance of these feature selection algorithms with the proposed algorithm for ccRCC (in addition to three other datasets; see Endothelial cell classification in different tumor types section for details) is shown in Figure 2(a). It can be seen that, although the proposed algorithm starts off slowly, after enough number of training examples (50 in our experiments), our algorithm performs as well as or better than the other algorithms. In our experiments, MRMR and T-test achieved optimum performance faster in all cases, although performance is matched by our active learning algorithm eventually. For the STS dataset, both MRMR and T-Test selected the same features and hence the classifier performance is identical for both these feature selection methods and the accuracy curve in orange indicates the performance of both MRMR and T-Test. The “slow start” of the active learning algorithm can be attributed to the fact that the proposed algorithm is working on the full training set using all the features and gradually makes decisions about the importance of features, whereas with the other algorithms the feature selection has already been performed and the classifier is working off the reduced feature set from the beginning. However, the proposed algorithm is much easier to use as the user can simply load the feature data and classify the data. The proposed algorithm achieved an estimated sensitivity of 0.942, specificity of 0.978, positive predictive value (PPV) of 0.883 and negative predictive value (NPV) of 0.989 (estimations used a model-based approach that accounts for correlation between nuclei due to clustered sampling). The results showed that the machine achieved the desired high level of specificity accompanied by excellent sensitivity. We also compared the performance of algorithms on the Wisconsin Breast Cancer (Diagnostic) dataset from the UCI Machine Learning Repository [29]. The features in this dataset are computed from a digitized image of a fine needle aspirate (FNA) of a breast mass and describe characteristics of the cell nuclei present in the image. The performance of the proposed algorithm is again similar to its performance on previous datasets, and in case of the UCI-II (UCI Wisconsin Diagnostic Dataset), the proposed algorithm outperforms the others (Fig. S2).

Although the performance of the algorithm in auto-select mode met expectations in all the datasets, we wanted to compare its classification performance in manual-select mode. To test this approach, the trainer selected the features to be used for EC classification (CD34 average, CD34 total, CD34 surround and elongated morphology) and allowed the algorithm to select only the informative training examples for which the user provided the labels. When compared with the auto-select mode, the algorithm with manual feature selection achieved a comparable specificity value (0.998) and PPV (0.99) but the performance degraded slightly with respect to sensitivity (0.93) and NPV (0.988). (Table 1).

**Table 1:** FARSIGHT-AL EC classification performance metrics.

	Sensitivity*		Specificity†		PPV▴		NPV•		Non-EC	EC
	Manual-select	Auto- select	Manual-select	Auto-select	Manual-select	Auto-select	Manual-select	Auto-select	(number)	(number)
**ccRCC20 image set**	0.93	0.942	0.998	0.978	0.990	0.883	0.988	0.989	7,037	1,323
**non-ccRCC image set**	0.965	0.941	0.999	0.997	0.982	0.934	0.998	0.998	13,070	515
**STS image set**	0.966	0.933	0.997	0.994	0.960	0.922	0.997	0.995	7,415	696
**K1735 image set**	0.925	0.896	0.998	0.996	0.942	0.911	0.997	0.996	16,553	691

*Sensitivity  =  True Positive/(True Positive + False Negative).

† Specificity  =  True Negative/(True Negative + False Positive).

▴ PPV  =  True Positive/(True Positive + False Positive).

• NPV  =  True Negative/(True Negative + False Negative).

**Figure 3:**
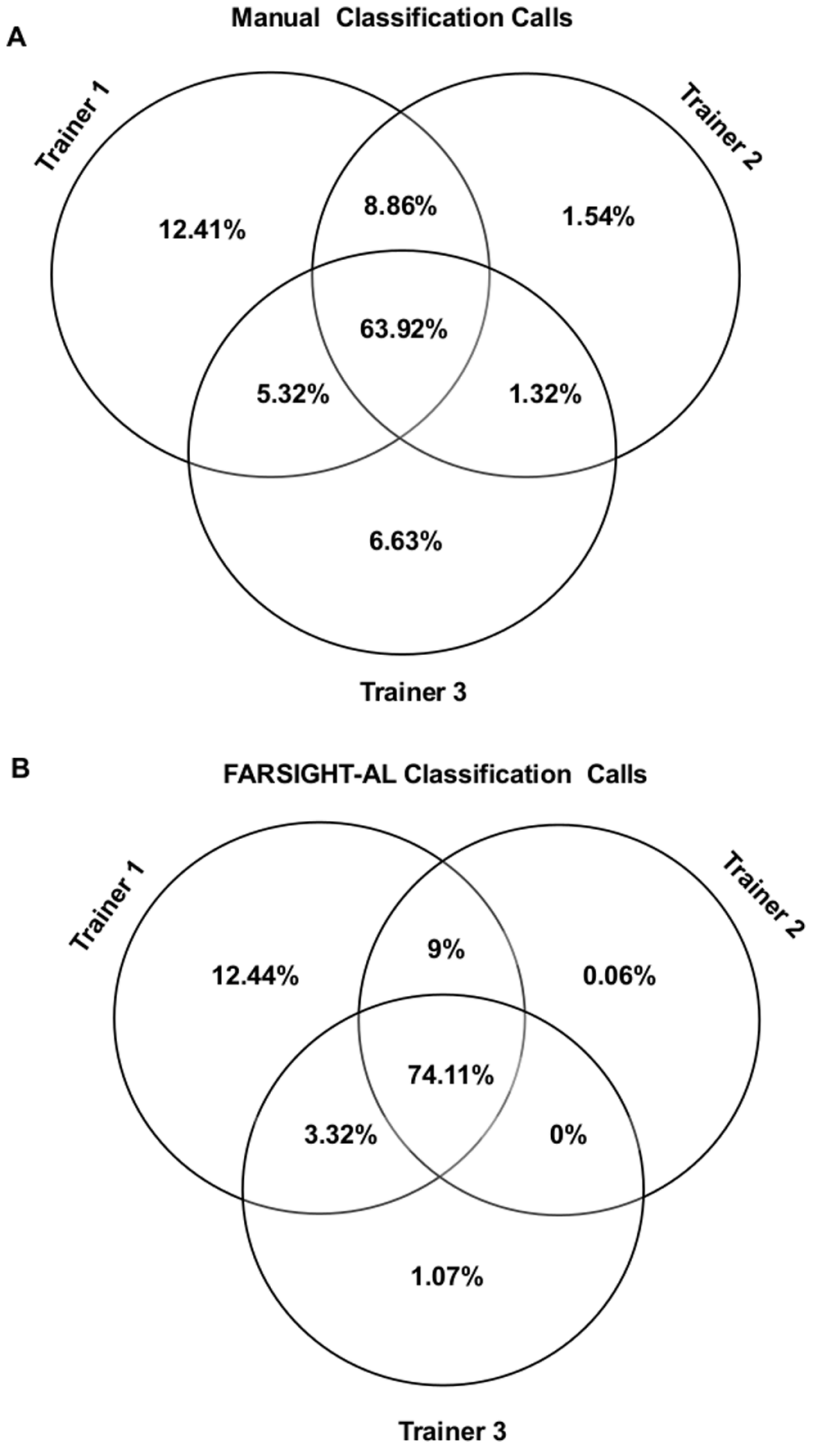
Venn diagrams for patterns of agreement in ccRCC20 dataset. Patterns of agreement between the classification calls made by each of the three experts and the FARSIGHT-AL classification models trained by them. The agreement was quantified based on a subset of cells in the ccRCC20 image set with cells that were classified as non-EC by all three experts being excluded in the analysis. (**A**) When classifying cells in this subset as EC or non-EC, the three experts agreed with one another only in ∼64% of the cases. (**B**) The agreement in FARSIGHT-AL classification models trained by each of the experts was ∼74%. Trainer 2 exhibits the least degree of “idiosyncrasy” in terms of classification calls and agrees better with trainer 1 than trainer 3.

Aware that human experts disagree frequently on which cells are EC in images, we evaluated how machine EC classification depended on its training. Two additional experts trained FARSIGHT-AL (in auto-select mode) to classify EC using the ccRCC20 image set and also personally classified EC in these images. Venn diagrams (Fig. 3) show the differences in EC classification by the three trainers and by the FARSIGHT-AL classification models created by them. For example, of cells classified as EC by at least one expert, ∼64% were classified as EC by all three, and ∼21% were classified as EC by only one. Trainer 1's manual classification calls agreed more with those of trainer 2 (∼73%) than with those of trainer 3 (∼70%). Interestingly, EC classification by each version of FARSIGHT-AL models agreed better among each other than the manual classification by their trainers. Once again, trainer 1's classification model agreed better with that of trainer 2 than trainer 3. Since classification models are mathematical rules, they tend to smooth out inconsistent classification calls, to which humans are susceptible, but preserve the overall preferences and idiosyncrasies of trainers. When agreement was quantified by pair-wise Cohen's kappa statistics (Table 2), the best agreement with each trainer's classification was achieved by the FARSIGHT-AL version that he/she trained (0.926 for trainer 1, 0.888 for trainer 2, 0.856 for trainer 3), meaning that the model generated by FARSIGHT-AL based on a trainer's selection conformed more closely to his/her preferences than any other trainer or model. Table S3 in File S1 shows the agreement matrices for EC classification with the FARSIGHT-AL models created by the three trainers.

**Table 2:** Quantifying trainer agreement evaluation using Kappa^ψ^ (κ) statistic.

	Trainer-1	FARSIGHT-AL (1)	Trainer-2	FARSIGHT-AL (2)	Trainer-3	FARSIGHT-AL (3)
**Trainer -1**	1.000	0.926	0.850	0.915	0.789	0.848
**FARSIGHT-AL(1)**	0.926	1.000	0.822	0.894	0.763	0.846
**Trainer -2**	0.850	0.822	1.000	0.888	0.826	0.860
**FARSIGHT-AL(2)**	0.915	0.894	0.888	1.000	0.808	0.901
**Trainer-3**	0.789	0.763	0.826	0.808	1.000	0.856
**FARSIGHT-AL(3)**	0.848	0.846	0.860	0.901	0.856	1.000

**^ψ^**Kappa Statistic is the ratio of observed agreement between raters to perfect agreement, controlling for agreement expected by chance alone. Refer to the “Kappa Statistics and Inter-Observer Agreements” sub-section in the Methods section to understand how Kappa values are calculated. The values in the above table reflect the agreement between the raters for EC classification in ccRCC20 image set. The diagonal values in the above table are all 1 as every rater agrees perfectly with himself/herself.

### Endothelial cell classification in different tumor types

With FARSIGHT-AL trained to classify EC in ccRCC tumors, we tested its ability to classify EC in other tumor types that had been similarly stained. Since the cell-type identifications of the three pathologists agreed reasonably, we used the classification models obtained by training against trainer 2's interpretations only. We compared models where features were auto-selected or manual-selected with human EC classification. EC were classified in 22 images containing a total of 13,585 cells taken from seven RCC tumors that were subtyped as non-clear cell (non-ccRCC image set) and included papillary type I and type II and chromophobe RCC. Comparison of the two models (Table 1) with human classification revealed that both the classification models achieved a high degree of specificity and sensitivity with the model with manual feature selection outperforming the automatic one slightly. The same models were also tested and shown to be able to classify EC with high accuracy in a set of 25 images with 8,111 cells taken from several different soft tissue sarcomas (STS image set) and a set of 20 images with 17,244 cells taken of stained K1735 murine melanoma tumors (K1735 image set). Thus, the classification model trained to classify EC in one type of human tumor can classify EC accurately in other tumor types, including those from other species. Table S2 in File S1 shows the agreement matrices with total cell numbers for each of the tumor types.

### Analysis of analyte expression in cells (analyte classification)

Accurate classification of EC in tumors using FARSIGHT-AL sets the stage for obtaining biologically meaningful information on these cells. Our interest in and the potential clinical significance of the biological activity of EC in tumors led us to train FARSIGHT-AL to determine their cell proliferative and signal transduction activities as reflected in EC expression of Ki67 and activated signaling intermediates, p-ERK and p-STAT3. The characteristic subcellular distribution and staining intensity of these analytes in immunostained ccRCC tumors dictated how EC were assessed for their presence: Ki67 and p-STAT3 are nuclear, and their expression was assessed by quantifying staining within nuclear boundaries drawn during segmentation; p-ERK is found in the nucleus, cytoplasm or both and was assessed by quantifying staining within a perimeter of two pixels (0.5 micron in images taken at 400X magnification) beyond nuclear boundaries. These factors and other distinctive characteristics of analyte immunostaining (see Fig. S3) led us to analyze datasets for analytes individually. The FARSIGHT user interface was modified to allow the user to navigate through hundreds of images and specify a threshold to classify cells as analyte-positive or analyte-negative.

**Figure 4:**
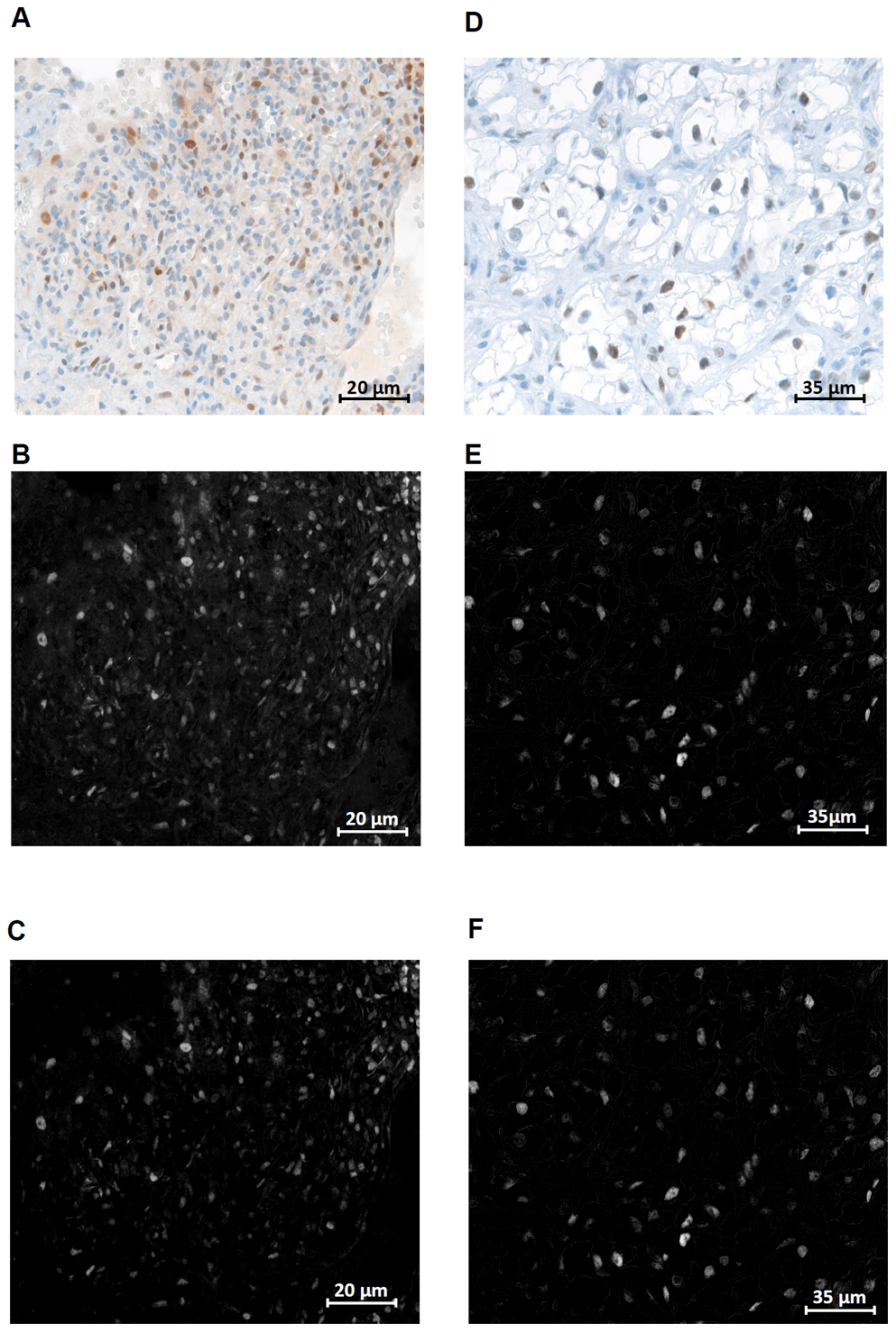
Background subtraction for analyte channels. To correct for background DAB staining in Ki67 and pSTAT3 (**A**), an extranuclear background subtraction algorithm was applied to the spectrally unmixed analyte channel (**B**), resulting in a new, “background-subtracted” analyte channel (**C**). Images with little to no DAB background (**D**) show almost no difference between the pre-subtraction analyte channel (**E**) and the background-subtracted analyte channel (**F**). Images in panels (A),(B), (C) display the same cropped region from a sample image. Images in panels (D),(E), (F) display the same cropped region from another sample image. The cropped regions are of different sizes which is reflected in the calibration bars.

An issue with quantifying analyte expression is the variability in analyte background signal (DAB staining) among different tumors and even among images from different regions of the same tumor. Selecting a threshold to classify analyte-positive cells without compensating for the background can result in many cells being incorrectly classified as analyte-positive. We observed this effect in Ki67 and p-STAT3 analyte channels with the p-ERK channel being relatively free of background staining effects. Therefore, both Ki67 and p-STAT3 channels were processed with a background subtraction algorithm that computes the average background signal (pixel intensity) and subtracts this value from the pixel intensities of the image. Figure 4 shows the results of the algorithm. It can be seen that when there is spurious background signal present in the spectrally unmixed analyte channel, the algorithm eliminates these effects (Fig. 4(A–C)) and does not modify analyte images without spurious background signal (Fig. 4(D–F)).

**Figure 5:**
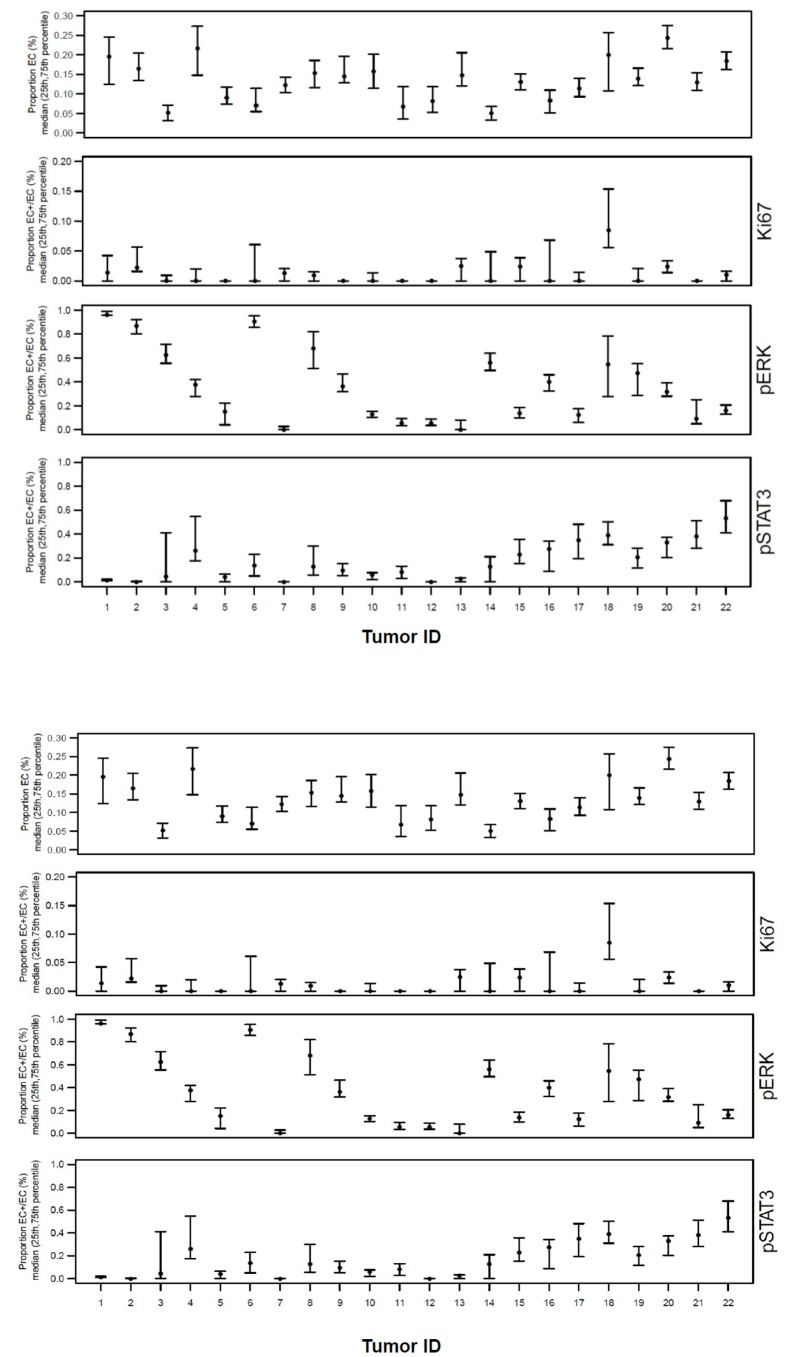
Analysis of analyte expression for 22 ccRCC tumors. (**A**) Results of endothelial cell (EC) classification and analysis of EC analyte expression performed on 22 ccRCC by Farsight-AL using the auto-select trainer 2 EC classification model and models for analyte classification specific to each analyte with operator-defined thresholds. Shown are the proportion of total cells classified as EC **(top)** and of EC staining positively for Ki67, p-ERK, and p-STAT3. Identifying codes for individual tumors are provided along the horizontal axis. EC proportions are represented by the median (solid circles) and the 75th and 25th percentile (upper and lower bars) values for the 10–12 image set collected for each tumor-analyte. (**B**) Similar results as in (A) for manual feature selection.

After performing background subtraction, cells classified as analyte-positive were the ones with analyte expression greater than the specified threshold. Trainer 2 manually classified every cell in the ccRCC20 image set as analyte- positive or negative and, treating this labeled set as ground truth, we validated the thresholding method against it. The method yielded excellent specificity and sensitivity (). Figure 5(A) shows plots of EC classification and analyte expression performed on an image set from 22 ccRCC tumors (10–12 images/tumor). Figure 5(B) shows the plots in the case where ECs were identified by the FARSIGHT-AL trainer 2 manual-select model. The auto-select and manual-select models yielded similar results, suggesting that FARSIGHT-AL auto-select model does not exhibit substantial bias towards classifying analyte-positive or -negative cells as EC. These results showed that the fraction of segmented cells identified as EC in the 22 tumors varied considerably (Figure 5; range 0.0386–0.3085, median 0.1294), but the median was significantly higher in these ccRCC tumors (p = 0.0015, Wilcoxon rank sum test) than in six papillary RCC tumors (range 0.0121–0.1126, median 0.0258) that we also studied (Fig. S4). In a tumor that had both ccRCC and papillary histologies, areas of the former had a significantly higher percentage of EC than areas of the latter. These results agree with the exceptional hypervascularity of tumors with clear cell histology and support the accuracy of FARSIGHT-AL EC analysis. Also notable is the wide variation in expression of EC activation antigens among tumors from different patients, with some showing comparatively low levels of the parameters measured. Substantial variation among different images from the same tumor also is evident for some tumors, suggesting regional heterogeneity in analyte expression and the possibility of “hot spots” of EC activation.

## Discussion

We present a software system with a sophisticated machine learning algorithm that is scalable and enables rapid, reproducible and accurate classification of cells with selective quantification of molecular analytes over subpopulations. It is responsive and searches features of hundreds of thousands of cells in a matter of seconds to present the user with the most informative cells for labeling. A critical requirement of any algorithm when dealing with human tumors is to adapt to the inherent variability present in the tissue samples, and our results on ccRCC tumors indicate that the algorithm performs very well in this regard. Moreover, the classifier model generated to identify EC in ccRCC performed similarly on data from different tumors from different species. Since cancer histopathology studies often examine similar cells in different tumor tissues, FARSIGHT-AL may save researcher time and effort in cell classification by creating representative cell type classification models that are broadly applicable and yield accurate results when the imaging protocols are similar. Our studies with multiple experts indicate that FARSIGHT-AL is malleable and learns the nuances of trainer interpretations. The algorithm is consistent and, as long as the initial examples and the labels provided by the trainer are the same, the algorithm queries for the same cells and yields the same final EC classification result; this contrasts with human EC classification, which often shows differences from occasion to occasion (data not shown).

An attractive feature of our system is the complete flexibility it provides to the user in terms of automation and robustness to user-selected settings. The auto-select mode requires minimal supervision from the user; this ability of the algorithm may be especially useful in cases where cell types of interest have not been stained with a specific marker and training relies heavily on morphological features that might not be easily specified. The system can be integrated in clinical workflow and used by researchers easily as it works without the need for careful parameter adjustments or specialized training. The auto-select mode allows development of accurate classification models when only images with the labeled cells are available. The pathologist labeling the cells need not be an expert in software, the software operator need not be expert in histopathology, and the two only have to communicate and grade results. All the algorithms and software have been integrated into the open-source FARSIGHT toolkit (www.farsight-toolkit.org).

We quantified antigen expression in cells of interest by determining the fraction of cells expressing a threshold level of antigen. FARSIGHT-AL permits antigen quantification in other ways. For example, the level of antigen expression in cells can be determined just as easily and expressed numerically as the mean or median or displayed graphically, resembling how flow cytometry results are usually displayed. Had we interest in an additional cell type in the tumors studied and could identify these cells with specificity, FARSIGHT-AL permits classification of additional cell types and allows characterization of two or more cell subpopulations using the same images. Similarly, additional analytes can be stained for and studied on the same slide and images, allowing examination of analyte co-expression by cells. These potentials of the “histocytometry” platform require development of corresponding multiplex immunostaining protocols and minor modification of existing FARSIGHT-AL algorithms.

The analytes chosen for initial study represent EC proliferation and signaling pathways activated by mitogens and found in angiogenic endothelium of mouse tumors and are expected to report endothelial activation and angiogenic activity in human tumors. Heterogeneity in their expression among patient ccRCC, a cancer known for hypervascularity and involvement of deregulated hypoxia response and angiogenesis in its pathogenesis, was not anticipated. On the other hand, finding differing levels of angiogenic activation and activity in patient tumors also should not surprise considering the variation in histopathology among ccRCC tumors, tumor size and stage at the time of resection, and clinical behavior and course among patients. However, another factor that may artifactually contribute to measurement variability must be considered; how patient specimens are handled prior to analysis. Tissue antigens are subject to enzymatic and oxidative degradation, and phospho-antigens may be particularly susceptible and labile. While we adopted stringent protocols for handling tumor slides (e.g. immunostaining was performed soon after cutting), information was unavailable about how tumors were handled prior to fixation and embedding. As quantitative analysis of biologically important antigens in patient cancers becomes feasible, it highlights the need for quality control and standards in the handling of clinical specimens to limit preanalytic variables.

Management of advanced RCC has been transformed by the advent of therapeutic agents that target components of the VEGF and other important EC signaling pathways [7]. Response to treatment with these agents, however, is variable and cannot be predicted for individuals on the basis of clinical, pathological or tumor genetic data. The ability of FARSIGHT-AL to identify and characterize EC in conventional histopathology specimens makes it possible to study EC biology in patient tumors and analyze and quantify activity of pathways that are either targeted or report on the activity of molecules targeted. We are awaiting clinical data about the patients whose tumors were characterized using FARSIGHT-AL to determine whether the measured parameters correlate with clinical outcome. This illustrates the opportunity for insight into the vascular biology of cancers of individual patients and potential discovery of rational predictive biomarkers of antiangiogenic therapeutic response.

## Materials and Methods

### Ethics statement

The Institutional Review Board of the University of Pennsylvania approved this study of renal cell carcinoma tumor samples previously collected by the Eastern Cooperative Oncology Group (ECOG) in their multi-institutional study protocol ECOG2804. Patients enrolled in ECOG2804 provided written informed consent for ECOG to collect tumor tissue previously removed.

### Tissue source

Human renal cell carcinoma specimens were obtained from the University of Pennsylvania's Department of Pathology & Laboratory Medicine's clinical paraffin archive and the Eastern Cooperative Oncology Group (ECOG) pathology core lab (Northwestern University, Chicago, IL) under IRB approval.

### Tissue staining

5 micron sections of formalin-fixed, paraffin embedded tumor tissues were deparaffinized and treated with citric acid monohydrate buffer (pH 6) at 75°C for 11 minutes prior to staining. The antibodies used for immunostaining included rabbit monoclonal anti-phospho-extracellular-signal-regulated-kinase (p-ERK), rabbit monoclonal anti-phospho-signal transducer and activator of transcription 3 (p-STAT3), mouse monoclonal anti-human Ki67 (Dako, Carpenteria, CA, USA), rabbit monoclonal anti-CD34 (Epitomics, Burlingame, CA, USA), mouse monoclonal anti-carbonic anhydrase 9 (CA IX) [30], and Cy3-conjugated anti-smooth muscle actin (SMA) (Sigma Life Science, St. Louis, MO, USA). p-ERK, p-STAT3, and Ki67 analytes were detected by immunohistochemistry with biotinylated species-specific secondary antibodies and avidin-linked horseradish peroxidase (HRP) (ABC kit, Vector Laboratories), followed by 3,3 diaminobenzidine (DAB, Vector Laboratories). CD34 and CA IX were detected by 3-step immunofluorescence using biotinylated species-specific secondary antibodies followed by fluorescently-labeled streptavidin conjugates (Alexa Fluor 488 and Alexa Fluor 647) (Invitrogen, Carlsbad, CA, USA). All slides were counterstained with hematoxylin after immunostaining.

Multiplex staining protocols were developed to stain slides with combinations of the above antibodies and chemicals to reveal cellular compartments and antigens that report on cell type and molecular analytes. A frequently used combination included staining for a molecular analyte with DAB, CD34 with Alexa Fluor 488, CA IX with Alexa Fluor 647, SMA with Cy3, and nuclei labeled in hematoxylin (Fig. S1).

### Tissue imaging

Multispectral images were captured using a Vectra® multispectral microscope and camera (PerkinElmer, Waltham, MA) at 400X magnification (8 bits/pixel depth) from 420 nm-720 nm at 20 nm wavelength intervals (brightfield mode) or 10 nm wavelength intervals (fluorescence mode). Nuance® software (PerkinElmer, Waltham, MA) was used to spectrally unmix the image cubes into individual channels corresponding to hematoxylin, DAB, and the fluorochromes (Alexa Fluor 488, Alexa Fluor 647, Cy3). Unmixing was based on the pure spectra of the respective chromogens and fluorochromes.

### Image processing

The spectral unmixing procedure results in multiple non-overlapping channels including a nuclear channel. We employed a hybrid segmentation algorithm that combines the algorithms proposed by Al-Kofahi et al [31] and Lin et al [32] to segment nuclei. The segmentation algorithm models the image intensities as a mixture of Poisson distribution and finds a threshold value based on minimum error thresholding. The initial binarization is refined using Graph Cuts [33]. The binarized image is then convolved with a Laplacian-of-Gaussian filter at different scales to detect seeds and compute initial segmentations (via local maximum clustering [34]) that are refined using a model based approach. In the refinement stage, adjacent fragments that share boundary pixels are merged and the scores of the merged nuclei are compared against the scores of individual fragmented nuclei. If the score of the merged nucleus is greater than the individual scores, then the nuclei are merged together in the final segmentation. The scores are evaluated using a training set of accurately segmented nuclei. Interested readers can refer to [31] and [32] for more details of the individual stages of the hybrid algorithm. For each segmented nucleus, we generate a range of “intrinsic” features that quantify their intensity, shape, size and chromatin texture (Table S1 in File S1) along with associative features that describe their association with multiple proteins across all the unmixed channels.

### Active learning for logistic regression

Each cell is denoted by a vector of cell measurements - 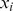, where the subscript i denotes the i^th^ cell. Considering a binary classification problem, the labels of the cells are denoted by 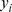 and +1 label indicates that the cell is an EC and a −1 label indicates that the cell is a non-EC. Following a standard logistic regression model notation, we can write 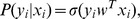



Where 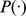 denotes the probability of an event, 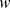 is a vector of classifier parameters that need to be estimated and 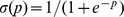 is the sigmoid function. For convenience, the intercept term has been accommodated by setting the first element of 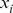
to always be 1. Assuming that the distribution of cell features are drawn independently and identically from the same distribution, the log-likelihood function can be written as 
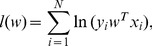
(1)where 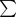
indicates summation over all 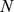 cells being analyzed and 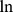 denotes the natural logarithm. In traditional supervised learning, the user provides a labeled set of examples to the algorithm with the goal of finding the value of 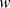 that maximizes **(1)**; i.e.,
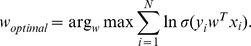



In the proposed method, training examples are selected *actively* based on the Fisher information matrix and this approach is called active learning. We denote the Fisher information matrix by 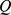 and by definition of the Fisher information matrix [35], we have
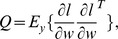
where 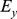 denotes the expectation operator with respect to the cell labels. It is well known that the inverse of the Fisher information matrix is a lower bound of the covariance matrix of the estimated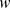. In particular, 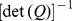
be a lower bound of the product of variances of the elements in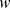. By selecting the cells that maximize 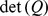, we can reduce the variances or uncertainty in the elements of 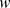. For logistic regression, 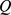 is given by 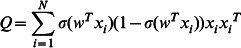



The selection of training examples proceeds in a sequential fashion where examples that maximize the determinant of the Fisher information criterion are queried for their labels; i.e., the algorithm queries the user for labls of those examples that maximize, in every iteration, the value of the expression 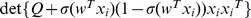. In the spirit of sequential optimal experiment design [36], we use the value of 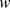 estimated from the labeled data selected until the current iteration. FARSIGHT-AL in auto-select mode uses L_1_-regularized logistic regression [37] which promotes sparse solutions and sets 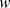 values of irrelevant features to zero thereby effectively selecting the features for classification. The maximization problem for training this classifier is, 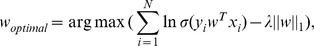
where 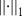 denotes the L_1_ norm and 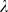 is a free parameter that can be used to control the degree of sparsity; i.e., a greater value of 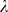 results in large number of 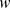 values being set to zero resulting in fewer features explaining the classification. To deal with the n on-differentiability of the L_1_- norm, we use the eps-L_1_ approximation to maximize the log-likelihood function. Finally, the Fisher Information matrix in auto-select mode is given by 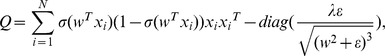
where 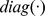
denotes a diagonal matrix and 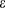
is a very small real number (of the order 10^−9^).

### Kappa statistics and inter-observer agreements

Cohen's Kappa (κ) [38] is a measure of agreement between two raters, ranging potentially from −1 to 1, with larger values indicating better agreement. It is defined as the ratio of observed agreement to perfect agreement, controlling for the level of agreement that would be expected by chance alone (i.e., as if the two ratings were independent). If we denote *P_o_* as the observed proportion of classification calls that are the same between two raters and *P_e_* as the proportion of classification calls that would be the same by chance given the observed data, then 
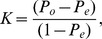



The numerator in this expression represents the proportion of observed agreement minus the agreement expected by chance assuming the fractions of observed positive ratings in the two classifiers, and the denominator represents the highest possible agreement minus the agreement expected by chance. If the raters agree as well as if they simply flipped coins i.e., completely randomly, *P_o_* will equal *P_e_* and κ will be zero. Although in general the interpretation of κ depends on the prevalence of a positive rating and the bias (the difference between raters in the proportion of positive ratings), a κ value of 0.6 or greater between raters is generally regarded as substantial agreement.

## Acknowledgments

Multispectral microscopy was performed in the Tumor Tissue and Biosample Bank (TTAB) facility, which is supported by the Department of Pathology & Laboratory Medicine and Abramson Cancer Center (P30 CA16520). We thank Jaromir Pastorek (Slovak Academy of Sciences, Bratislava, Slovak Republic) for M75 anti-CA IX antibody [30].

## Supporting Information

Figure S1 **Multispectral image of a multiplex-stained ccRCC tumor with results of spectral unmixing.** A clear cell renal cell carcinoma (ccRCC) slide was stained for an analyte (Ki67) and nuclei using chromogens DAB (brown) and hematoxylin (blue), respectively, and for antigens that mark different cells types (CD34, SMA, CA IX) using different fluorochromes. After spectral unmixing, the brightfield image (**A**) yielded hematoxylin (**B**) and DAB (**C**) chromogen channels, which were used for nuclear segmentation and analyte determination, respectively. After spectral unmixing, the fluorescent image (**D**) yielded the Alexa Fluor 488 (**E**), Cy3 (**F**) and Alexa Fluor 647 (**G**) channels, which were used to stain CD34 (endothelial cell), SMA (pericyte) and CA IX (tumor cell) antigens, respectively.
(TIF)

Figure S2 **Comparison of FARSIGHT-AL performance with other feature selection algorithms for UCI machine learning breast cancer datasets.** Mean classification accuracy of 25 independent simulations plotted as a function of number of training examples for different automated feature selection algorithms including FARSIGHT AL (blue lines) on the UCI Breast Cancer Datasets. FARSIGHT-AL selected 50 training examples sequentially based on the increase in information gain whereas logistic regression was used to classify examples after feature selection by PCA (green), T-Test (purple), MRMR (Orange). Standard Logistic regression (red) with no feature selection performs poorly compared to other algorithms. The bars indicate standard error of the mean of classification accuracy.
(TIF)

Figure S3 **Subcellular distribution of different analytes.** The diversity in the staining patterns of different analytes is illustrated in the above figure. The nuclear stain appears blue in color whereas the analyte stain appears brown. Ki67 (**A**) and pSTAT3 (**B**) are predominantly nuclear bound whereas pERK (**C**) IS found in both the cytoplasmic and nuclear regions.
(TIF)

Figure S4 **Comparison of ccRCC and non-ccRCC tumors for hypervascularity.** The whisker plot shows the proportion of EC as a percentage of total number of cells in 22 ccRCC tumors (left) and 6 non-ccRCC tumors (right). In both the panels, the dot and the whiskers follow standard notation i.e., the dot indicates the median value of the proportion of cells and the top and bottom whiskers indicate the 25th and 75th percentile. Visual inspection of these plots suggests that the proportion of EC is higher in the ccRCC case. Comparison of the median values from each group using the Wilcoxon rank sum test revealed statistically significant differences between the groups with a p-value of 0.0015.
(TIF)

File S1 **File containing Tables S1–S4.**
(DOC)

## References

[1] Mcallister SS, Weinberg RA (2010) Tumor-host interactions: a far-reaching relationship. J Clin Oncol 28: 4022–8. 20644094 10.1200/JCO.2010.28.4257

[2] Ferrara N (2005) Kerbel (2005) RS Angiogenesis as a therapeutic agent. Nature 438: 967–74. 16355214 10.1038/nature04483

[3] Weis SM (2011) Cheresh (2011) DA Tumor Angiogenesis: molecular pathways and therapeutic strategies. Nature Medicine 17: 1359–1370. 10.1038/nm.2537 22064426

[4] Hurwitz H, Fehrenbacher L, Novotny W, Cartwright T, Hainsworth J, et al (2004) Bevacizumab plus irinotecan, fluorouracil, and leucovorin for metastatic colorectal cancer. N Engl J Med 350: 2335–42. 15175435 10.1056/NEJMoa032691

[5] Sandler A, Gray R, Perry MC, Brahmer J, Schiller JH, et al (2006) Paclitaxel-Carboplatin Alone or with Bevacizumab for Non-Small-Cell Lung Cancer. N Engl J Med 355: 2542–50. 17167137 10.1056/NEJMoa061884

[6] Eberhard A, Kahlert S, Goede V, Hemmerlein B, Plate KH, et al (2000) Heterogeneity of angiogenesis and Blood Vessel Maturation in Human Tumors: Implications for Antiangiogenic tumor therapies. Cancer Research 60: 1388–1393. 10728704

[7] Yao X, Qian CN, Zhang ZF, Tan MH, Kort EJ, et al (2007) Two distinct types of blood vessels in clear cell renal cell carcinoma have contrasting prognostic implications. Clin Cancer Res 13: 161–9. 17200351 10.1158/1078-0432.CCR-06-0774

[8] Sharma S, Sharma MC, Sarkar C (2005) Morphology of angiogenesis in human cancer: a conceptual overview, histoprognostic perspective and significance of neoangiogenesis. Histopathology 46: 481–489. 15842629 10.1111/j.1365-2559.2005.02142.x

[9] Lassoued W, Murphy D, Tsai J, Oueslati R, Thurston G, et al (2010) Effect of VEGF and VEGF Trap on vascular endothelial cell signaling in tumors. Cancer Biol Ther 10: 1326–33. 21079419 10.4161/cbt.10.12.14009 PMC3047090

[10] Murphy DA, Makkonen S, Lassoued W, Feldman MD, Carter C, et al (2006) Inhibition of tumor endothelial ERK activation, angiogenesis, and tumor growth by sorafenib (BAY43-9006). Am J Pathol 169: 1875–85. 17071608 10.2353/ajpath.2006.050711 PMC1780219

[11] Li L, Kaelin WG Jr (2011) New Insights into the biology of renal cell carcinoma. Hematol Oncol Clin North Am 25: 667–86. 21763962 10.1016/j.hoc.2011.04.004 PMC3161447

[12] Négrier S, Raymond E (2012) Antiangiogenic treatments and mechanisms of action in renal cell carcinoma. Invest New Drugs 30: 1791–801. 21573959 10.1007/s10637-011-9677-6

[13] Bjornsson CS, Lin G, Al-Kofahi Y, Narayanaswamy A, Smith KL, et al (2008) Associative image analysis: A method for automated quantification of 3D multi-parameter images of brain tissue. J Neurosci Methods 170: 165–78. 18294697 10.1016/j.jneumeth.2007.12.024 PMC2700351

[14] Liao X, Carin L (2009) Migratory logistic regression for learning concept drift between two data sets with application to UXO sensing. Geoscience and Remote Sensing, IEEE Transactions on 47: 1454–1466.

[15] Tong S, Koller D (2002) Support vector machine active learning with applications to text classification. The Journal of Machine Learning Research 2: 45–66.

[16] Muslea I, Minton S, Knoblock CA (2000) Selective sampling with redundant views AAAI/IAAI

[17] McCallum A, Nigam K (1998) Employing EM and Pool-Based Active Learning for Text Classification. ICML 98: 350–358.

[18] Zhu X, Lafferty J, Ghahramani Z (2003) Combining active learning and semi-supervised learning using gaussian fields and harmonic functions. ICML workshop on the continuum from labeled to unlabeled data in machine learning and data mining.

[19] Guo Y, Greiner R (2007) Optimistic Active-Learning Using Mutual Information. Proceedings of the International Joint Conference on Artificial Intelligence 7: 823–829.

[20] Liu Y (2004) Active learning with support vector machine applied to gene expression data for cancer classification. Journal of chemical information and computer sciences 44 (6) 1936–1941. 15554662 10.1021/ci049810a

[21] Xu Z, Akella R, Zhang Y (2007) Incorporating diversity and density in active learning for relevance feedback. Advances in Information Retrieval. Springer Berlin Heidelberg 246–257.

[22] Rubens N, Kaplan D, Sugiyama M (2011). Active learning in recommender systems Recommender Systems Handbook, Springer US 735–767.

[23] Doyle S, Madabhushi A (2010). Consensus of ambiguity: theory and application of active learning for biomedical image analysis. Pattern Recognition in Bioinformatics, Springer Berlin Heidelberg 313–324.

[24] Doyle S, Monaco J, Feldman MD, Tomaszewski J, Madabhushi A (2009) A class balanced active learning scheme that accounts for minority class problems: Applications to histopathology. OPTIMHisE Workshop (MICCAI).

[25] Al-Kofahi Y, Lassoued W, Grama K, Nath SK, Zhu J, et al (2011) Cell-based quantification of molecular biomarkers in histopathology specimens. Histopathology 59: 40–54. 21771025 10.1111/j.1365-2559.2011.03878.x PMC3142095

[26] Bennett KP, Embrechts MJ (2003) An optimization perspective on kernel partial least squares regression. Nato Science Series sub series III computer and systems sciences 190: 227–250.

[27] Student. (1908). The probable error of a mean. Biometrika, 1–25.

[28] Peng H, Long F, Ding C (2005) Feature selection based on mutual information criteria of max-dependency, max-relevance, and min-redundancy. Pattern Analysis and Machine Intelligence, IEEE Transactions on, 27(8): 1226–1238. 10.1109/TPAMI.2005.159 16119262

[29] Bache K, Lichman M (2013) UCI Machine Learning Repository [http://archive.ics.uci.edu/Irvine, CA: University of California, School of Information and Computer Science.

[30] Chia SK, Wykoff CC, Watson PH, Han C, Leek RD, et al (2001) Prognostic significance of a novel hypoxia-regulated marker, carbonic anhydrase IX, in invasive breast carcinoma. J Clin Oncol 19: 3660–8. 11504747 10.1200/JCO.2001.19.16.3660

[31] Al-Kofahi Y, Lassoued W, Lee W, Roysam B (2010) Improved automatic detection and segmentation of cell nuclei in histopathology images. IEEE Trans Biomed Eng 57: 841–52. 19884070 10.1109/TBME.2009.2035102

[32] Lin G, Chawla MK, Olson K, Barnes CA, Guzowski JF, et al (2007) A multi-model approach to simultaneous segmentation and classification of heterogeneous populations of cell nuclei in 3D confocal microscope images. Cytometry A 71: 724–36. 17654650 10.1002/cyto.a.20430

[33] Boykov Y, Veksler O, Zabih R (2001) Fast approximate energy minimization via graph cuts. Pattern Analysis and Machine Intelligence, IEEE Transactions on 23: 1222–1239.

[34] Wu X, Chen Y, Brooks BR, Su YA (2004) The Local Maximum Clustering Method and Its Application in Microarray Gene Expression Data Analysis. EURASIP J. Appl. Signal Process 1: 53–63.

[35] Cover TM, Thomas JA (2006) Elements of Information Theory. New York:Wiley-Interscience.

[36] Fedorov VV (1972) Theory of optimal experiments. New York:Academic Press.

[37] Lee S, Lee H, Abbeel P, Ng AY (2006) Efficient L-1 Regularized Logistic Regression. In Proceedings of the National Conference on Artificial Intelligence 21: 401.

[38] Cohen J (1960) A coefficient of agreement for nominal scales. Educational and psychological measurement 20: 37–46.

