# Supplementary figures and images for: An Active Learning Approach for Rapid Characterization of Endothelial Cells in Human Tumors
Source: PLoS One. 2014 Mar 6;9(3):e90495. doi: 10.1371/journal.pone.0090495 (PMC3946171; doi:10.1371/journal.pone.0090495)

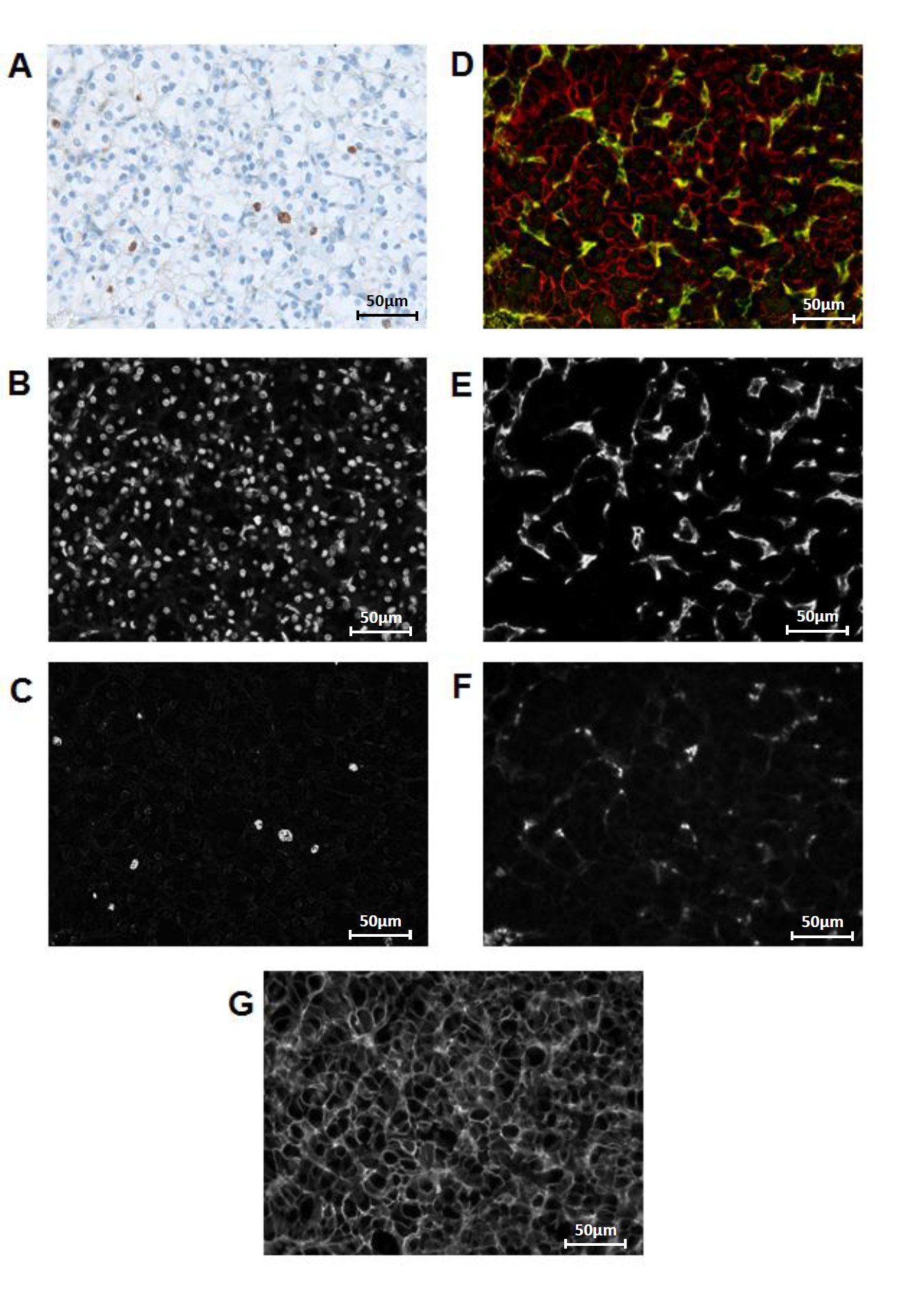

Supplement: Figure S1 — Multispectral image of a multiplex-stained ccRCC tumor with results of spectral unmixing. A clear cell renal cell carcinoma (ccRCC) slide was stained for an analyte (Ki67) and nuclei using chromogens DAB (brown) and hematoxylin (blue), respectively, and for antigens that mark different cells types (CD34, SMA, CA IX) using different fluorochromes. After spectral unmixing, the brightfield image (A) yielded hematoxylin (B) and DAB (C) chromogen channels, which were used for nuclear segmentation and analyte determination, respectively. After spectral unmixing, the fluorescent image (D) yielded the Alexa Fluor 488 (E), Cy3 (F) and Alexa Fluor 647 (G) channels, which were used to stain CD34 (endothelial cell), SMA (pericyte) and CA IX (tumor cell) antigens, respectively. (TIF) [file pone.0090495.s001.tif]

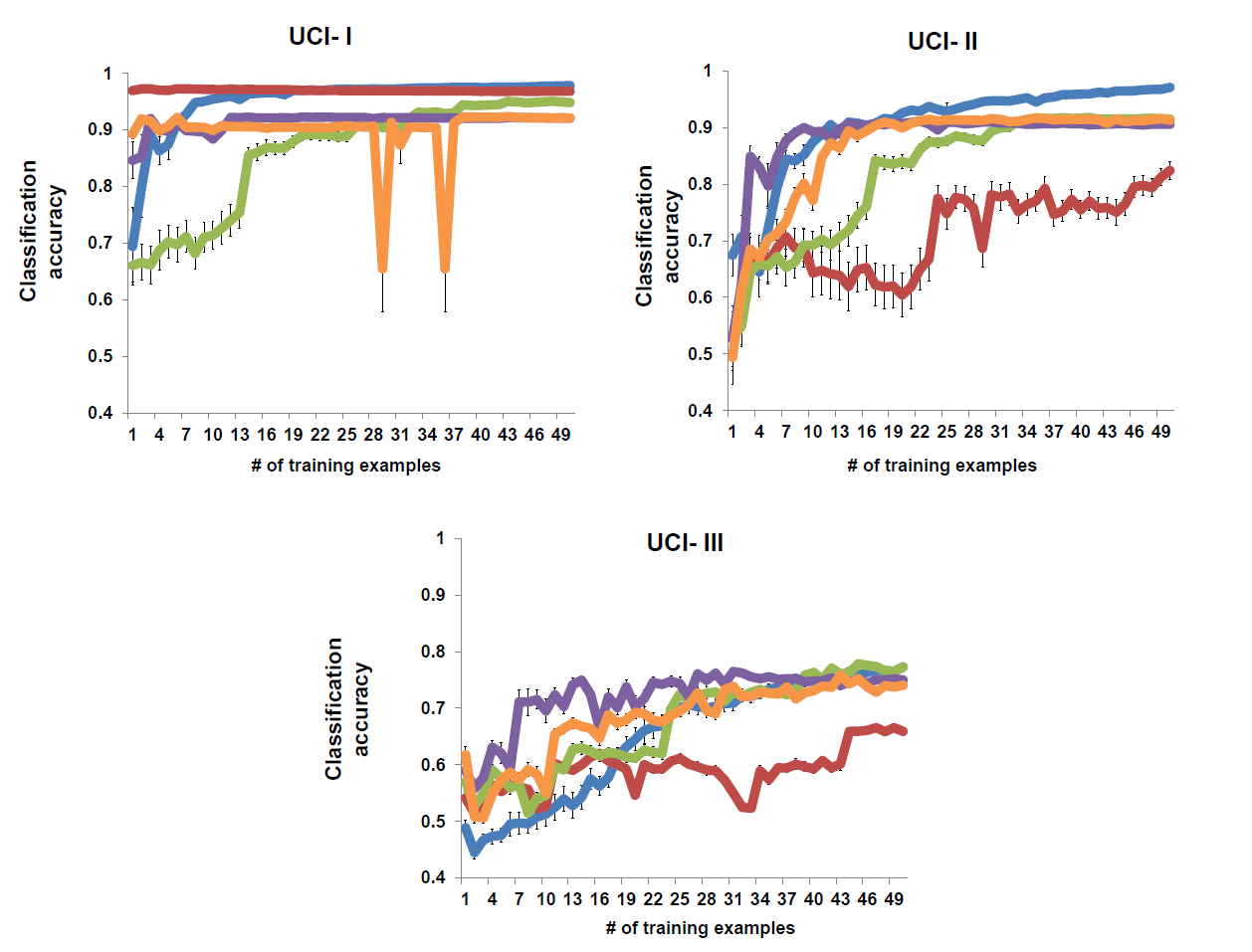

Supplement: Figure S2 — Comparison of FARSIGHT-AL performance with other feature selection algorithms for UCI machine learning breast cancer datasets. Mean classification accuracy of 25 independent simulations plotted as a function of number of training examples for different automated feature selection algorithms including FARSIGHT AL (blue lines) on the UCI Breast Cancer Datasets. FARSIGHT-AL selected 50 training examples sequentially based on the increase in information gain whereas logistic regression was used to classify examples after feature selection by PCA (green), T-Test (purple), MRMR (Orange). Standard Logistic regression (red) with no feature selection performs poorly compared to other algorithms. The bars indicate standard error of the mean of classification accuracy. (TIF) [file pone.0090495.s002.tif]

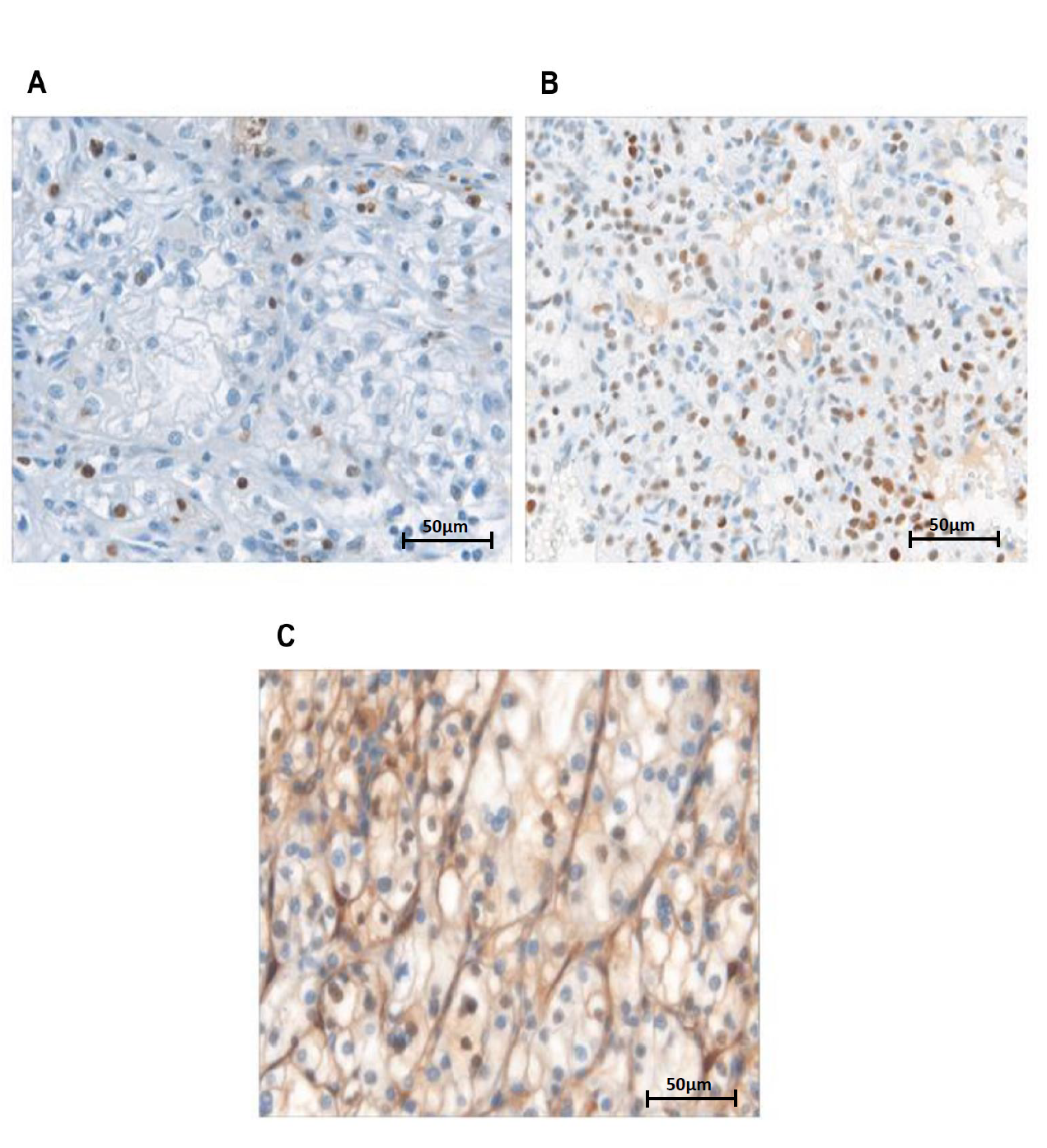

Supplement: Figure S3 — Subcellular distribution of different analytes. The diversity in the staining patterns of different analytes is illustrated in the above figure. The nuclear stain appears blue in color whereas the analyte stain appears brown. Ki67 (A) and pSTAT3 (B) are predominantly nuclear bound whereas pERK (C) IS found in both the cytoplasmic and nuclear regions. (TIF) [file pone.0090495.s003.tif]

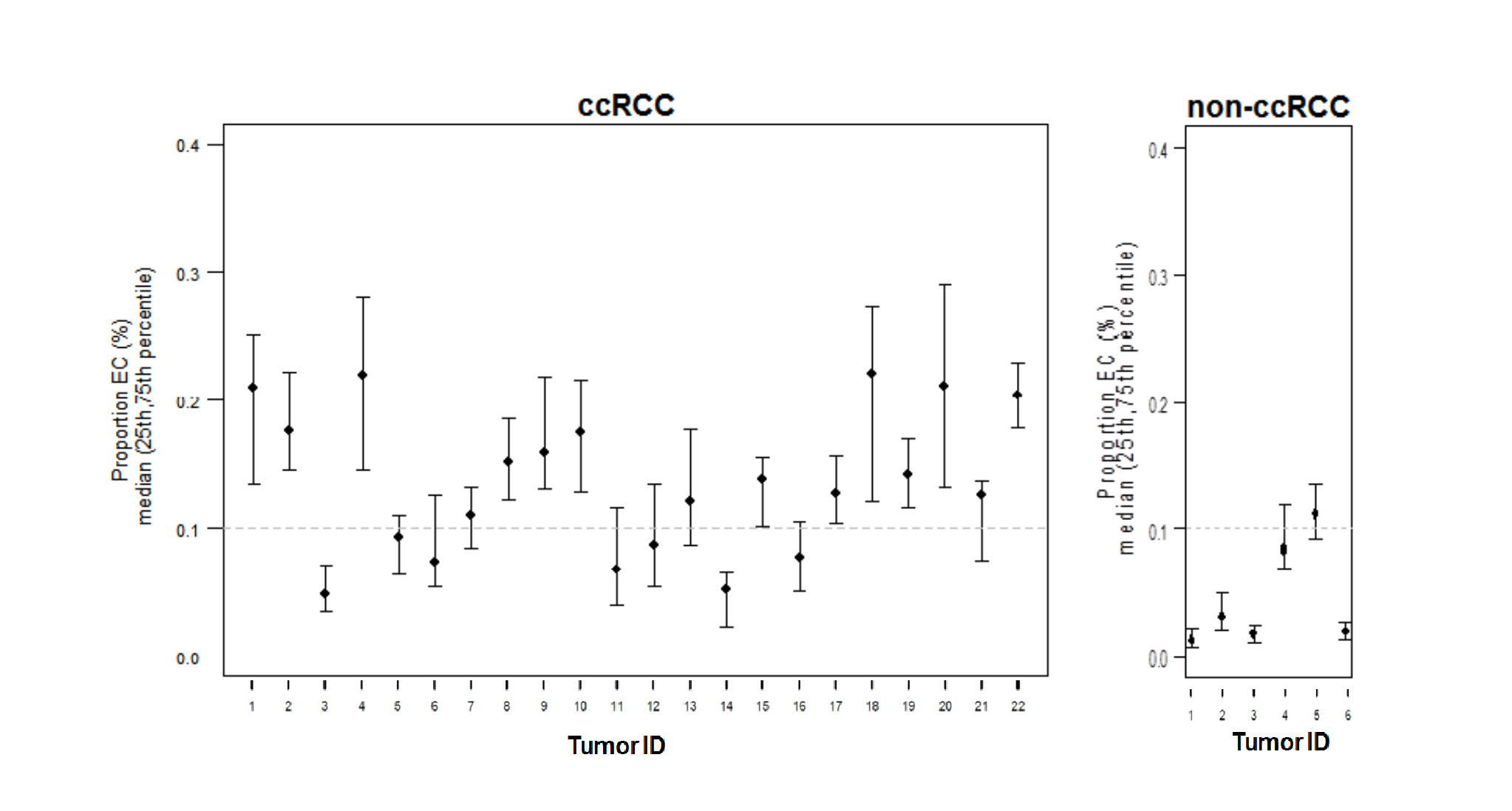

Supplement: Figure S4 — Comparison of ccRCC and non-ccRCC tumors for hypervascularity. The whisker plot shows the proportion of EC as a percentage of total number of cells in 22 ccRCC tumors (left) and 6 non-ccRCC tumors (right). In both the panels, the dot and the whiskers follow standard notation i.e., the dot indicates the median value of the proportion of cells and the top and bottom whiskers indicate the 25th and 75th percentile. Visual inspection of these plots suggests that the proportion of EC is higher in the ccRCC case. Comparison of the median values from each group using the Wilcoxon rank sum test revealed statistically significant differences between the groups with a p-value of 0.0015. (TIF) [file pone.0090495.s004.tif]
