# Supplementary material for: An Active Learning Approach for Rapid Characterization of Endothelial Cells in Human Tumors
Source: PLoS One. 2014 Mar 6;9(3):e90495. doi: 10.1371/journal.pone.0090495 (PMC3946171; doi:10.1371/journal.pone.0090495)
Supplement: File S1 — File containing Tables S1–S4. (DOC) [file pone.0090495.s005.doc]

# Supplementary Information

**Table S**1. Description of “intrinsic” features

| **Feature Name** | | | **Description** | |
| --- | --- | --- | --- | --- |
| Volume | | | Number of pixels in the cell | |
| Integrated Intensity | | | Sum of all pixel intensities inside the cell | |
| Eccentricity | | | Ratio of the distance between the foci of the best-fit hyper-ellipsoid to the length of its major axis | |
| Elongation | | | Ratio of the major axis length to minor axis length of the best-fit hyper-ellipsoid | |
| Orientation | | | Angle between the major axis of the best-fit hyper-ellipsoid and origin | |
| Bounding Box Volume | | | Number of pixels in the bounding box of the cell | |
| Mean | | | Average intensity of pixels in the cell | |
| Minimum | | | Minimum intensity of pixels in the cell | |
| Maximum | | | Maximum intensity of pixels in the cell | |
| Sigma | | | Standard deviation of intensity of pixels in the cell | |
| Variance | | | Variance of intensity of pixels in the cell | |
| Surface Gradient | | | Average gradient value of surface pixels | |
| Interior Gradient | | | Average gradient value of interior pixels | |
| Surface Intensity | | | Average intensity value of surface pixels of the cell | |
| Interior Intensity | | | Average intensity value of interior pixels of the cell | |
| Intensity Ratio | | | Ratio of surface intensity to interior intensity | |
| Convexity | | | Ratio of surface area to area of the convex hull | |
|  | Radius Variation | Standard deviation of distance from surface pixels to the centroid | |  |
|  | Surface Area | Number of pixels on the surface of the cell | |  |
|  | Shape | Ratio of surface pixels to total pixels that represents the compactness of the cell | |  |
|  | Shared Boundary | Ratio of edge sharing cell pixels to total number of edge pixels | |  |
|  | T-Energy | Measure of homogeneity calculated from the gray-level co-occurrence matrix | |  |
|  | T-Entropy | Measure of randomness in image intensities calculated from the gray-level co-occurrence matrix | |  |
|  | Inverse Difference Moment | Measure of homogeneity calculated from the gray-level co-occurrence matrix | |  |
|  | Inertia | Measure of contrast calculated from the gray-level co-occurrence matrix | |  |
|  | Cluster Shade | Measure of skewness in the pixel intensities in the cell calculated from the gray-level co-occurrence matrix | |  |
|  | Cluster Prominence | Measure of skewness in the pixel intensities in the cell calculated from the gray-level co-occurrence matrix | |  |
|  | Zernike Moments | Image moments describing the shape characteristics of the cell based on Zernike Polynomials | |  |

Description of “Intrinsic features” that describe their shape, intensity, size and chromatin texture. These features are computed for every cell detected by the segmentation algorithm.

**Table S2 (A-C). Agreement matrices for different tumor types**

**(A**)

| **Non-ccRCC** | | **Trainer** | | |
| --- | --- | --- | --- | --- |
|  |  | **Non-EC** | **EC** | **Total** |
| **FARSIGHT-AL** | **Non-EC** | 13,060**^ψ^** | 21**^Δ^** | 13,081 |
|  | **EC** | 10**^π^** | 494**^ϯ^** | 504 |
|  | **Total** | 13,070 | 515 | 13,585 |

| **STS** | | **Trainer** | | |
| --- | --- | --- | --- | --- |
|  |  | **Non-EC** | **EC** | **Total** |
| **FARSIGHT-AL** | **Non-EC** | 7,380 **^ψ^** | 25 **^Δ^** | 7,405 |
|  | **EC** | 35 **^π^** | 671 **^ϯ^** | 706 |
|  | **Total** | 7,415 | 696 | 8,111 |

**(B)**

**(C)**

| **K1735** | | **Trainer** | | |
| --- | --- | --- | --- | --- |
|  |  | **Non-EC** | **EC** | **Total** |
| **FARSIGHT-AL** | **Non-EC** | 16,514 **^ψ^** | 56 **^Δ^** | 16,570 |
|  | **EC** | 39**^π^** | 635**^ϯ^** | 674 |
|  | **Total** | 16,553 | 691 | 17,244 |

**Ψ** - Number of true negatives i.e., cells classified as non-EC by both the expert and the model,

**Δ** - Number of false negatives i.e., cells classified as non-EC by the model and as EC by the expert,

**π** - Number of false positives i.e., cells classified as EC by the model and non-EC by the expert and

**ϯ** - Number of true positives i.e., cells classified as EC by both the expert and the model.

**Table S3. Performance Metrics for FARSIGHT analyte classification**

| **Metric** | **Population** | **Analyte** | | |
| --- | --- | --- | --- | --- |
|  |  | **Ki67*** | **pSTAT3*** | **pERK** |
| **Sensitivity** | **All Cells** | 0.850 | 0.803 | 0.968 |
|  | **EC-only^Ϯ^** | 0.865 | 0.849 | 0.981 |
| **Specificity** | **All Cells** | 0.997 | 0.989 | 0.988 |
|  | **EC-only^Ϯ^** | 1.00 | 0.981 | 0.992 |
| **PPV** | **All Cells** | 0.949 | 0.955 | 0.979 |
|  | **EC-only^Ϯ^** | 1.00 | 0.940 | 0.993 |
| **NPV** | **All Cells** | 0.991 | 0.947 | 0.981 |
|  | **EC-only^Ϯ^** | 0.996 | 0.948 | 0.979 |

**^Ϯ^**FARSIGHT also allows for quantifying analyte expression over all cells or over chosen sub-populations like EC only (as above) and classify them as analyte positive or negative.

* Ki67 and pSTAT3 analyte channels were processed with background subtraction before computing their analyte expression levels.

**Table S4 (A-C). Agreement matrices of FARSIGHT-AL classification models**

**(A**)

|  |  | **Trainer 1** | | |
| --- | --- | --- | --- | --- |
|  |  | **Non-EC** | **EC** | **Total** |
| **FARSIGHT**  **AL-1** | **Non-EC** | 6,999**^ψ^** | 215**^Δ^** | 7,214 |
|  | **EC** | 11**^π^** | 1,135**^ϯ^** | 1,146 |
|  | **Total** | 7,010 | 1,350 | 8,360 |

|  |  | **Trainer 2** | | |
| --- | --- | --- | --- | --- |
|  |  | **Non-EC** | **EC** | **Total** |
| **FARSIGHT**  **AL-2** | **Non-EC** | 7,007 **^ψ^** | 101 **^Δ^** | 7,108 |
|  | **EC** | 30 **^π^** | 1,222 **^ϯ^** | 1,252 |
|  | **Total** | 7,307 | 1,323 | 8,360 |

**(B)**

**(C)**

|  |  | **Trainer 3** | | |
| --- | --- | --- | --- | --- |
|  |  | **Non-EC** | **EC** | **Total** |
| **FARSIGHT**  **AL-3** | **Non-EC** | 6,766 **^ψ^** | 90 **^Δ^** | 6,856 |
|  | **EC** | 11**^π^** | 1,493**^ϯ^** | 1,504 |
|  | **Total** | 6,777 | 1,583 | 8,360 |

Numbers in the tables indicate the number of cells that are classified as EC or non-EC and compared against the interpretation of each of the three experts training the model. The superscript **Ψ** indicates the number of true negatives i.e., cells classified as non-EC by both the expert and the model, **Δ** indicates the number of false negatives i.e., cells classified as non-EC by the model and as EC by the expert, **π** indicates the number of false positives i.e., cells classified as EC by the model and non-EC by the expert and **ϯ** indicates the number of true positives i.e., cells classified as EC by both the expert and the model .
